# P392: Reprocessing of manicures and pedicures material in salons in Brazil

**DOI:** 10.1186/2047-2994-2-S1-P392

**Published:** 2013-06-20

**Authors:** JL Garbaccio, AC Oliveira, AO de Paula

**Affiliations:** 1 Nursing, Federal University of Minas Gerais, Belo Horizonte, Brazil

## Introduction

In Brazil, due to the habit of removing the cuticles of nails, bleedings are common and, without appropriate sterilization of instruments, it can be an important factor of contamination by viruses of hepatitis B and C.

## Objectives

The objective of this study was to verify the knowledge and the adherence to the reprocessing techniques of manicures/pedicures from Belo Horizonte, Brazil.

## Methods

This Survey included a random sample of 200 professionals, older than 18, in 200 beauty salons. A questionnaire was applied (Jul/12-Jan/13) to obtain information of demographics, the cleanness and the sterilization procedures. The Ethics Committee of the Federal University of Minas Gerais approved the study.

## Results

All professionals interviewed were women, with an average age of 30, 10 years of experience (11%), working 8 hours per day (57%), employed less than a year in that salon (34%). As for education, 55% had completed high school, 54% had done some training course in the field, however 38% became manicure/pedicure by their own initiative. Moreover 76% had never received any training in biosafety. The respondents chose incorrect statements in the questions about the knowledge of the decontamination concepts (65%), cleaning (53%) and disinfection (51%), stating that such processes kill all microorganisms. Regarding sterilization, the correct answers achieved a higher score 66%, however 20% defined sterilization as disinfection and cleaning. With regards to decontamination practice, only 1% stated to submerge in enzymatic detergent and only 39% referred to wash with water/soap. All respondents confirmed that they sterilize the materials after use. The dry heat (oven) was the process most cited (65%), followed by moist heat (autoclave) with 31% and 34% revealed not believe that the method used in the salon can sterilize the material. In the knowledge questions, participants chose autoclave (65%) as ideal process of sterilization followed by oven (53%). About the exposure time of the materials to the sterilizing agent, only 16% do it in the right way. The temperature of heat exposure was correct in 34%.

## Conclusion

It was noticed a lack of knowledge and incorrect practice of the recommendations and techniques what doesn´t guarantee a correct material reprocessing among manicures/pedicures.

## Funding Agent

Fapemig PPM nº 00340-11

## Disclosure of interest

None declared

